# Protecting Older Adult Residents in Care Facilities Against Influenza and COVID-19 Using the Influenza Communication, Advice and Reporting (FluCARE) App: Prospective Cohort Mixed Methods Study

**DOI:** 10.2196/38080

**Published:** 2023-03-13

**Authors:** Emma Quinn, Kai Hsun Hsiao, Travers Johnstone, Maria Gomez, Arun Parasuraman, Andrew Ingleton, Nicholas Hirst, Zeina Najjar, Leena Gupta

**Affiliations:** 1 Public Health Unit Sydney Local Health District Sydney Australia; 2 Faculty of Medicine and Health School of Public Health University of Sydney Sydney Australia

**Keywords:** web app, digital health, influenza, COVID-19, outbreak, monitoring, disease control, infection spread, infection control, detect, aged care, elderly, elderly population, older adult, long term care, care home, AFC, LTC, nursing home, retirement home, mobile application, health application, mHealth, care facility, online training, health impact, feasibility, efficacy, satisfaction, prevention, disease spread, notification

## Abstract

**Background:**

Early detection and response to influenza and COVID-19 outbreaks in aged care facilities (ACFs) are critical to minimizing health impacts. The Sydney Local Health District (SLHD) Public Health Unit (PHU) has developed and implemented a novel web-based app with integrated functions for online line listings, detection algorithms, and automatic notifications to responders, to assist ACFs in outbreak response. The goal of the Influenza Outbreak Communication, Advice and Reporting (FluCARE) app is to reduce time delays to notifications, which we hope will reduce the spread, duration, and health impacts of an influenza or COVID-19 outbreak, as well as ease workload burdens on ACF staff.

**Objective:**

The specific aims of the study were to (1) evaluate the acceptability and user satisfaction of the implementation and use of FluCARE in helping ACFs recognize, notify, and manage influenza and COVID-19 outbreaks in their facility; (2) identify the safety of FluCARE and any potential adverse outcomes of using the app; and (3) identify any perceived barriers or facilitators to the implementation and use of FluCARE from the ACF user perspective.

**Methods:**

The FluCARE app was piloted from September 2019 to December 2020 in the SLHD. Associated implementation included promotion and engagement, user training, and operational policies. Participating ACF staff were invited to complete a posttraining survey. Staff were also invited to complete a postpilot evaluation survey that included the user Mobile Application Rating Scale (uMARS) measuring app acceptance, utility, and barriers and facilitators to use. An issues log was also prospectively maintained to assess safety. Survey data were analyzed descriptively or via content analysis where appropriate.

**Results:**

Surveys were completed by 31 consenting users from 27 ACFs. FluCARE was rated 3.91 of 5 overall on the uMARS. Of the 31 users, 25 (80%) would definitely use FluCARE for future outbreaks, and all users agreed that the app was useful for identifying influenza and COVID-19 outbreaks at their facilities. There were no reported critical issues with incorrect or missed outbreak detection. User training, particularly online training modules, and technical support were identified as key facilitators to FluCARE use.

**Conclusions:**

FluCARE is an acceptable, useful, and safe app to assist ACF staff with early detection and response to influenza and COVID-19 outbreaks. This study supports feasibility for ongoing implementation and efficacy evaluation, followed by scale-up into other health districts in New South Wales.

## Introduction

Influenza [1] and, more recently, COVID-19 [2,3] outbreaks in aged care facilities (ACFs) have had significant health impacts on both older adult residents and staff. They also place an additional workload burden on staff managing the outbreak [4]. In Australia, reported attack rates for influenza in older adult residents of ACFs are around 14%, with hospitalization and death rates estimated to be 10.5% and 4.2%, respectively, depending on the season [5]. A review that included 49 influenza outbreaks in ACFs across 19 countries found that residents experienced a median attack rate of 33%, hospitalization rate of 14%, and death rate of 6.5% [6]. A systematic review of COVID-19 outbreaks in ACFs across 49 studies from 4 continents reported a mean single-facility attack rate of 45% (95% CI 32%-58%), hospitalization rate of 37% (95% CI 35%-39%), and case fatality rate of 23% (95% CI 18%-28%) [7]. Older adult residents in ACFs are particularly vulnerable to the health impact of these infectious diseases due to their overall frailty, close-quarter living arrangements, shared caregivers, and frequent visitors coming into the facility [3,4].

Prevention measures such as vaccinations are usually the ideal approach to minimizing the risk and impacts of either an influenza [4] or COVID-19 [8] outbreak in the aged care setting. However, influenza vaccine efficacy can be suboptimal for older adults due to immune senescence and comorbidities [9], and poor vaccine coverage in aged care staff can exacerbate the introduction of influenza into ACFs [10]. Furthermore, although research evidence has demonstrated that COVID-19 vaccine confers protection against severe disease, efficacy wanes over time (6 months), by 20% to 30% [11]. Therefore, outbreaks still occur in ACFs with high resident vaccination coverage rates [5].

Consequently, early outbreak recognition, notification, and response will continue to be critical to minimizing the health impacts of these outbreaks for older adult residents in aged care [3,5]. In New South Wales (NSW), Australia, local public health units (PHUs) are responsible for providing advice and support to ACFs for influenza [12] and COVID-19 [13] outbreak management and monitoring under the Australian national guidelines. However, delays in recognition and notification to the PHU occur [14,15], and these delays are associated with increased attack rates, outbreak duration, and mortality [5]. In Australia, for every 24-hour delay in time to PHU notification for an influenza outbreak in an ACF, there is an associated increase in outbreak duration of 0.674 days [5].

Several factors may contribute to delays in outbreak recognition, including complexities for ACF staff in interpreting national guidelines and criteria for respiratory outbreaks [14,15], atypical symptom presentation of influenza and COVID-19 in older adults [16,17], and the time required for laboratory confirmation, which may be due to fear of negative publicity and high workload, especially once an outbreak has been declared. Several studies have suggested training and education for ACF staff are the best strategies to improve recognition and understanding of the importance of the timeliness of outbreak recognition and response [18,19]. However, published evidence on the effectiveness of such educational programs is limited. Alternatively, there is potential to create and use new innovative technological tools that prompt ACF staff in outbreak recognition, notification, and management processes [5,20].

The Sydney Local Health District (SLHD) PHU has developed and implemented a novel web-based app accessible on both mobile and desktop devices to address some of the key factors contributing to delays in outbreak management. The app’s integrated features include an online line list, which is a table that contains key information about each case (resident) in an outbreak, with each row representing a case and each column representing a variable such as demographic and clinical information. In addition to line lists, the app also has algorithms for outbreak detection, automatic notifications to responders (ie, nurses and managers within facilities and PHU staff) for influenza, and response checklists and resources. Registered users within facilities (ie, nurses or managers) actively use the app on a daily basis to enter, manage, and report on influenza and COVID-19 cases according to national public health guidelines [12,13]. The app’s algorithms automatically detect an outbreak based on criteria within the national guidelines [12], subsequently notifying responders to support the timeliness of the response. The app has dashboards to display key summary outbreak metrics at the facility and PHU level, as well as reporting functionality, which enables the facility or PHU to download a line list on any day, and outbreak metrics for further analysis. A full description of the app functions and technical design is provided in an earlier article [21]. The goal of the Influenza Outbreak Communication, Advice and Reporting (FluCARE) app is to reduce time delays to notifications, which we hope will reduce the spread, duration, and health impacts of an influenza or COVID-19 outbreak, as well as ease workload burdens on ACF staff. The FluCARE app was implemented in the SLHD as a pilot program starting in September 2019, with its outbreak surveillance and management functions targeted at influenza outbreak management. However, with the evolution of the COVID-19 pandemic and the recognition of the app’s comparable utility for COVID-19 surveillance in ACFs, FluCARE underwent further rapid development to incorporate COVID-19 functionalities in March 2020 (based on ACF and PHU staff feedback).

The purpose of this paper was to describe the evaluation of the acceptability, safety, and utility of FluCARE based on the experience of ACF users during this pilot period. This will inform further program development and scale-up. The specific aims of the study were to:

1. Evaluate the acceptability and user satisfaction of the implementation and use of FluCARE in helping ACFs recognize, notify, and manage influenza and COVID-19 outbreaks in their facility.
2. Identify the safety of FluCARE and any potential adverse outcomes of using the app.
3. Identify any perceived barriers or facilitators to the implementation and use of FluCARE from the ACF user perspective.

## Methods

### FluCARE Pilot Program

The FluCARE pilot program ran from September 2019 to December 2020 across 62 ACFs in the SLHD [22]. Any ACF in the district was able to voluntarily register for a FluCARE account. Accounts were validated by the PHU to ensure appropriate access to the app. Users were typically nurse managers or infection prevention and control managers. All users were required to complete FluCARE training via a face-to-face workshop or online modules prior to using FluCARE.

The FluCARE app and pilot had been widely promoted to ACFs through face-to-face workshops, teleconference updates, email communications, and telephone calls. Dedicated email and telephone support was provided to ACFs for registration, user training, app utilization, and troubleshooting. A detailed description of the package of activities to support app implementation is provided in an earlier article [21]. Evaluation tools, training modules, and user manuals for ACFs were also updated from March 2020 to support use and evaluation of the additional COVID-19 functionalities.

### Ethics Approval

Ethical approval was granted by the Ethics Review Committee of the SLHD (protocol number: X19-0157). All registered ACF users in the SLHD were invited to participate in the study and were emailed a “participant information statement” outlining the details of the study and a consent form to sign and return to the research team.

### Study Participants

One or more staff members from ACFs in the SLHD were eligible for the study based on completion of voluntary registration to FluCARE. The eligible staff members were generally the person(s) responsible for submitting line lists to the PHU, for example, a care manager, registered nurse, infection control officer, or facility manager. Participation in the study was not a requirement for using FluCARE. Those who consented to participate but did not subsequently complete the required training modules or workshop to use FluCARE were excluded from the study. Study participants were invited to complete the training evaluation survey immediately following their completion of training and the FluCARE evaluation survey at the end of the pilot period in December 2020.

### Scope of the Evaluation

**Figure 1:**
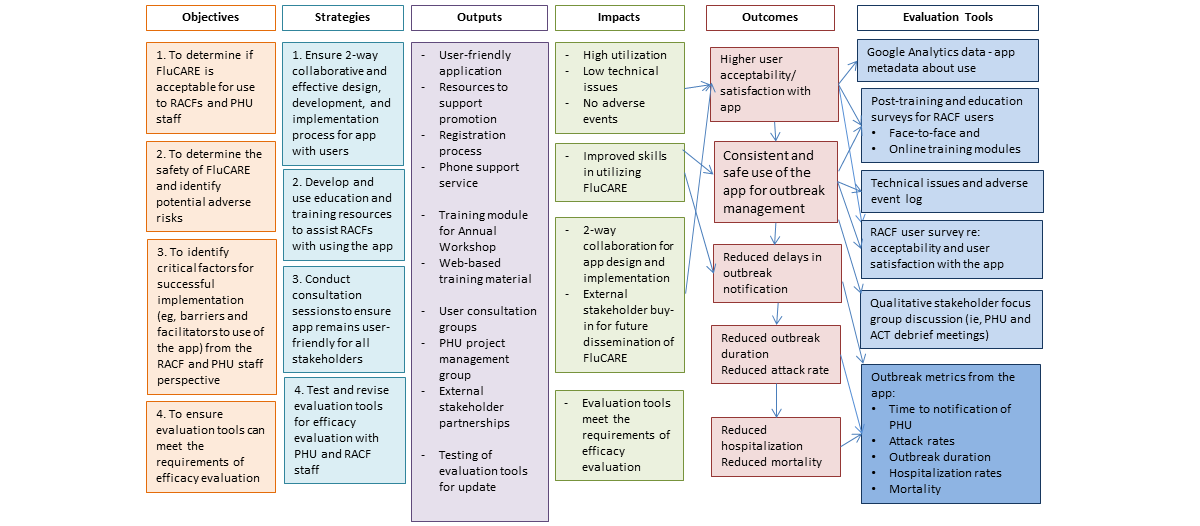
Logic frame model for the pilot evaluation of the Influenza Communication, Advice and Reporting (FluCARE) app. ACT: access care team; PHU: public health unit; RACF: residential aged care facility.

The logic frame model for the FluCARE pilot program and evaluation is shown in Figure 1. The inputs and activities conducted in the design, development, and implementation of FluCARE have been published elsewhere [21]. In the following sections, we describe the evaluation tools used to measure app acceptability, utility, and safety, as well as the barriers and facilitators to implementation of the app.

### Measures

#### Training Evaluation Survey

FluCARE training was evaluated using a semistructured survey instrument containing questions in 3 domains: (1) training content or delivery, (2) user experience with the training, and (3) confidence in using FluCARE posttraining. Survey questions within each domain were adapted for the face-to-face workshops and for the online training modules to be applicable to each delivery modality. Workshop participants were invited to complete the posttraining evaluation survey online through REDCap [23], an online secure database system available in the SLHD for research purposes, or via a paper form that PHU staff entered into REDCap at a later stage. Evaluation surveys for the online training were built into the Moodle (a learning management system) platform for completion at the end of the modules [24]

#### ACF User App Evaluation Survey

The ACF user app evaluation survey was the main evaluation instrument to gather information from ACF users on their engagement and satisfaction with the FluCARE app via the validated user Mobile Application Ratings Scale (uMARS) survey [25]. Information was also sought on barriers and facilitators to adoption of the app within their facility and organization based on a framework of implementation factors suggested in the literature [26,27]. This semistructured instrument collected data in 5 main domains: (1) participant demographics, (2) self-rated workload assessment and use of app, (3) uMARS, (4) barriers and facilitators, and (5) use of resources and support. The instrument was piloted with 5 ACF users to ensure face validity; content validity was assessed by cross-checking the survey questions against the domains and subdomains of the reported factors representing barriers and facilitators to use of apps within the literature [26,27]. Study participants were invited to complete the survey via a scheduled telephone interview at the end of the study period (ie, December 2020). The survey instrument was sent to participants at least one week before their interview date to assist with preparation and recall of use, as well as engagement with FluCARE.

#### Issues Log

As part of the monitoring and evaluation of the app’s safety, a log was set up to collect information on any technical issues or adverse events that occurred during the pilot program, including date of issue, name of notifier, whether identified by PHU or ACF, the function or task it was related to in FluCARE, description of issue, comments on resolution, and resolution date (if possible). PHU staff completed this log every time an issue was raised by either ACF or PHU staff and actively followed up with the SLHD Information Communication Technology (ICT) services to ensure resolution was achieved where possible.

#### Outbreak Metrics From the App

The FluCARE app was designed to be able to report influenza and COVID-19 outbreak data to PHU and ACF users as a dashboard functionality, with the option of downloading a situation report (with aggregate outbreak metrics for a specific influenza or COVID-19 outbreak) as well as a line list report (with detailed line listing data for every resident or staff member with symptoms or signs of influenza or COVID-19).

### Data Analysis

#### Training Evaluation Survey

Survey results from the workshops and online training modules were separately analyzed and summarized with descriptive statistics in SAS Enterprise Guide, Version 8.2 (SAS Institute).

#### ACF User App Evaluation Survey

During the interview, the senior research officer (MG) directly entered the interview data into the REDCap database, including checking each response with the participant to ensure reliability of transcription. Quantitative survey data were analyzed descriptively in SAS Enterprise Guide, Version 8.2, and content analysis was performed on responses to open-ended questions using Microsoft Excel.

#### Issues Log

Descriptive and content analyses were performed on quantitative and qualitative data as described in the previous section to report on duration to resolution and common categories of issues, respectively.

## Results

### Participants

**Figure 2:**
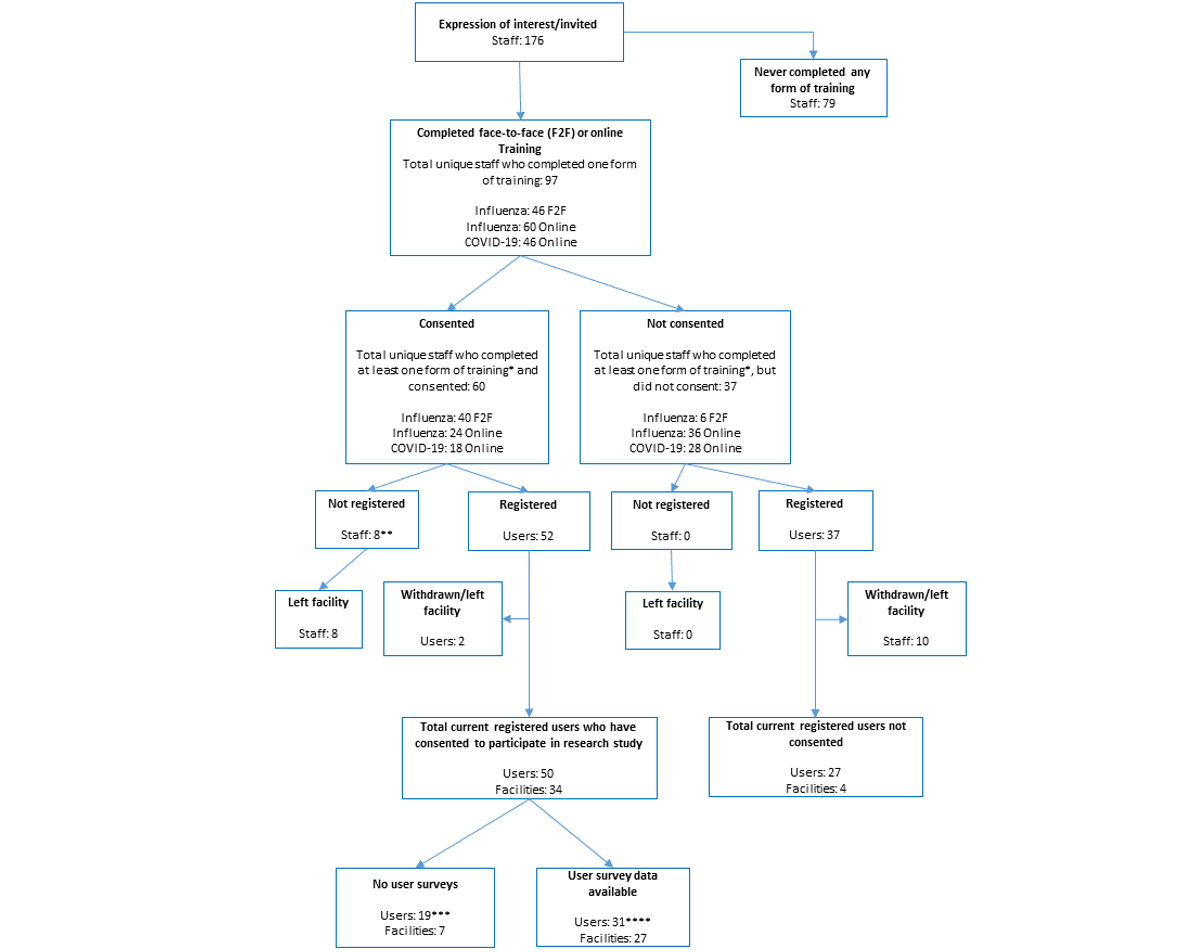
Study flowchart. Staff refers to people who were trained or consented but not registered. Users refers to staff who were trained.*Users can complete more than one form of training; **included posttraining surveys only; ***metadata/Google Analytics data available only, no other evaluation data; ****all evaluation data.

As shown in Figure 2, 176 ACF staff were invited to participate in this evaluation study of the FluCARE pilot; 52 (29.5%) users from 34 facilities consented and were eligible (ie, completed training and app registration) to participate in this study (Figure 2). Of these users and facilities, 31 (62%) users from 27 (79%) facilities completed the postpilot app evaluation survey.

### ACF User Education and Training Surveys

Of the 46 users completing face-to-face workshop training for FluCARE use in influenza outbreak management, 40 (87%) participated in the study (Figure 2). Of the 60 and 46 users completing online training for FluCARE use in influenza and COVID-19 outbreak management, respectively, 24 (40%) and 18 (39%; for a total of 42 respondents) participated (Figure 2). Of all workshop respondents, all (40/40, 100%) agreed that the content of the workshop was helpful. The vast majority (38/40, 95%) agreed that the workshop was engaging, all (40/40, 100%) felt more confident in using FluCARE, and 92% (35/38) said that the content was interesting. A vast majority of online training respondents agreed that the online training module content was easy to follow (40/42, 95%), useful (40/42, 95%), and of sufficient depth (39/42, 93%). Overall, 95% (40/42) of respondents felt confident in using FluCARE at the end of the training. Users rated the online training courses 7.5 of 10. The survey responses from app users who attended or completed training are shown in Table 1.

**Table 1:** Survey responses from app users who attended face-to-face workshops (n=40) or completed online training modules (n=42).

Evaluation indicator^a^ and corresponding survey items			Survey response^b^, n (%)
**Training content and delivery, n (%)**			
	**Face-to-face workshop and survey**		
		The workshop was well-organized.	38 (95)
		The workshop was well-facilitated.	40 (100)
		The pace of the training during the workshop was about right.	39 (97)
		The content of the workshop was helpful for using FluCARE^c^ for the purpose of influenza outbreak management.	40 (100)
	**Online training modules and survey**		
		The videos are easy to understand.	41 (98)
		The content was easy to follow.	40 (95)
		The time to complete the module is [appropriate].	33 (79)
		The online training module presents useful information on how to use FluCARE.	40 (95)
		Course topics are dealt with in sufficient depth.	39 (93)
		The design of the online training module lets me learn at my own pace.	37 (88)
**User experience with training**			
	**Face-to-face workshop and survey, n (%)**		
		Participants were encouraged to ask questions.	39 (98)
		The workshop was engaging.	38 (95)
		The content of the workshop was interesting.	37 (92)
	**Online training modules and survey**		
		What would you rate the training out of 10? mean (range)	7.48 (3-10)
**Confidence in using FluCARE, n (%)**			
	**Face-to-face workshop and survey**		
		I feel more confident in knowing how to use FluCARE to help my facility detect, notify, and respond to an influenza outbreak.	40 (100)
	**Online training modules and survey**		
		I feel confident in using FluCARE.	40 (95)
		I feel comfortable in applying the skills learned in this course.	40 (95)

^a^All semistructured questions were answered on a scale from strongly agree, agree, neutral, disagree, to strongly disagree.

^b^Survey responses in the table correspond to the users reporting strongly agree or agree only.

^c^FluCARE: Influenza, Communication, Advice and Reporting.

### ACF User Surveys

Of the 50 users (from 34 facilities) who consented to participate in our study, 31 (62%) users from 27 (79%) facilities completed the postpilot app evaluation survey (Figure 2); 6 (19%) respondents reported not using FluCARE for outbreak reporting during the study period. The reasons given included no outbreak to report, access issues with the app, or issues with delineation of users to complete line listing. Therefore, 25 (81%) respondents had used FluCARE for outbreak surveillance and reporting. Their key characteristics are described in Table 2.

Respondents reported having a median of 1 (range 0-4) influenza outbreak at their facility in the past 5 years. The majority of respondents reported using the FluCARE app to record a single respiratory virus case (23/25, 92%) or a suspected case of COVID-19 on the line list (19/25, 76%) during the pilot period (multichoice answer), whereas 68% (17/25) reported using FluCARE to record multiple respiratory virus cases (including cases of influenza-like illness [ILI] or confirmed influenza). The survey results found that 32% (8/25) of respondents used the app to access resources and information. As shown in Table 3, FluCARE was helpful in identifying the first few cases of ILI (15/25, 60% agreement) or in determining whether the facility had a COVID-19 situation to monitor (16/25, 64% agreement). Other survey results revealed that 84% (21/25) of respondents reported that it was easy or very easy to submit the daily line listings via the app.

User ratings of FluCARE on the uMARS scale are summarized in Table 4. Of the 4 domains in the uMARS, the highest scores were for the quality of information (mean 4.53, SD 0.33), followed by the aesthetics of the app (mean 4.25, SD 0.43). FluCARE was rated 3.91 of 5 overall on the uMARS.

Of the survey respondents, 68% (17/25) reported that they would “definitely” recommend the app to other people, and 80% (20/25) reported that they would “definitely” use the app for future influenza and COVID-19 outbreaks. However, only 28% (7/25) of respondents would definitely pay for the app. There was a high level of self-reported agreement (agree and strongly agree) that FluCARE would (1) improve the ability of the facility to recognize both influenza (24/25, 96%) and COVID-19 outbreaks (24/25, 96%), (2) assist the facility in knowing which actions to take once an outbreak has been recognized (19/25, 76%), (3) reduce the time to notify the PHU (23/25, 92%), (4) reduce time in talking to stakeholders in outbreak management (22/25, 88%), (5) make the submission of line listing easier (25/25, 100%), and (6) be useful and beneficial for influenza (25/25, 100%) and COVID-19 (25/25, 100%) outbreak management.

Regarding technical barriers or facilitators to use of the app, there was a high level of agreement (21/25, 84%) from respondents that the app made it easy to (1) complete outbreak detection, notification, and management tasks and (2) access it within the local IT network and that respondents were (3) confident of the information and (4) security within the app. However, respondents were less agreeable (13/25, 52%) about how easy the app was to access outside their organization (ie, from home).

There was a high level of agreement from respondents that (1) the education and training provided sufficient support to users to learn how to use the app (25/25,100%), (2) the technical support provided by the PHU to users was adequate and supported use (25/25, 100%), and (3) they had sufficient time to practice using the app in routine practice (21/25, 84%). However, respondents were less likely to agree (7/25, 28%) that they felt involved in the design of the app.

At least two-thirds of respondents agreed that the use of FluCARE was prioritized by their team and organization for use in outbreak management (22/25, 88%) and they had sufficient organizational support to use the app (18/25, 72%). However, there was less agreement (10/25, 40%) from respondents that there was accountability within their organization if FluCARE was not used and that there were incentives from their organization to use the app.

**Table 2:** Characteristics of survey respondents (n=25) and their facilities (n=27) participating in the postpilot app evaluation survey.

Characteristics		Results
**Number of residents or staff, median (range)**		
	Total number of residents	62 (23-130)
	Total number of high-care residents	50 (3-130)
	Total number of staff at the facilities	72 (24-170)
Number of outbreaks in last 5 years (2016-2020) at your facility, median (range)		1 (0-4)
**Influenza outbreaks during the pilot period (September 2019 to Dec 2020), n^a^**		
	Single influenza	2
	Single influenza-like illness	21
**COVID-19 outbreaks during the pilot period (September 2019 to Dec 2020), n^a^**		
	Close monitoring	19
	Potential outbreaks	3

^a^Total number of situations at the 12 facilities (from 27) that reported outbreak situations.

**Table 3:** Survey results (n=25) indicating how FluCARE was used during the pilot program.

Survey item	Yes, n (%)	No, n (%)	Unsure, n (%)	N/A^a^, n (%)
FluCARE^b^ helped identify the first few cases of influenza-like illnesses at their facility.	15 (60)	2 (8)	2 (8)	6 (24)
FluCARE helped recognize that the facility potentially had an influenza outbreak.	12 (48)	1 (4)	2 (8)	10 (40)
FluCARE helped recognize that the facility potentially had a COVID-19 situation.	16 (64)	2 (8)	0 (0)	7 (28)
FluCARE automatically notified you that a potential influenza outbreak was occurring in your facility.	7 (28)	0 (0)	0 (0)	18 (72)
FluCARE helped you identify the appropriate next steps to manage and control the outbreak within your facility.	3 (12)	1 (4)	0 (0)	21 (84)

^a^Respondents worked in a facility that did not have a situation (influenza or COVID-19) that met the survey item statement.

^b^FluCARE: Influenza Communication, Advice and Reporting.

**Table 4:** Survey results (n=25) based on the user Mobile Application Rating Scale (uMARS).

Survey item^a^			Results, mean (SD)
**Engagement score**			
	Entertainment: Is the app entertaining or interesting to use?	3.2 (0.7)	
	Customization: Does the app allow you to customize settings and preferences?	1.9 (1.0)	
	Interactivity: Does the app allow user input, all the users to provide feedback, and contain prompts?	2.7 (1.0)	
	Target group: Is the app content appropriate for the target audience?	4.4 (0.6)	
Total engagement score			3.1 (0.5)
**Functionality score**			
	Performance: How accurately/fast do app features and components work?	3.3 (0.8)	
	Ease of use: How easy is it to learn to use the app?	4.1 (0.7)	
	Navigation: Does navigation through the app make sense to you?	4.0 (0.8)	
Total functionality score			3.8 (0.5)
**Aesthetics**			
	Layout: Is the arrangement and size of buttons, icons, menus, etc appropriate?	4.3 (0.6)	
	Graphics: How high is the quality/resolution of graphics for buttons, icons, menus, etc?	4.3 (0.8)	
	Visual appeal: How does the app look?	4.2 (0.5)	
Total aesthetics score			4.3 (0.4)
**Quality of information**			
	Quality of information: Is the app content correct, up-to-date, well-written, etc?	4.3 (0.5)	
	Quality of information: Is the information in the app comprehensive but concise?	4.6 (0.6)	
	Visual information: Are the visual concepts in the app clear, logical, and correct?	4.3 (0.6)	
	Credibility of source: Does the information in the app come from a credible source?	4.9 (0.3)	
Total quality of information score			4.5 (0.3)
Overall score			3.9

^a^uMARS survey items are all scored out of 5, from 1 (poor) to 5 (excellent).

### Issues Log

From September 2019 to December 2020, there were 27 entries in the issue log. Of these, 21 were reported by PHU staff, and 6 were reported by ACF staff. The majority of recorded issues were related to access (18/27, 67%). Access issues were primarily technical, with incidents of generic page not found or internal server errors on attempting to access FluCARE. Most of these were resolved on the same day, either spontaneously or with ICT assistance. However, on one occasion, restoration of access took 20 days, which required implementation of our Business Continuity Plan with manual procedures and provision of downtime packs and communications to ACFs. Three access issues were related to delays in receiving the 2-factor authentication code. Other issues on the log related to the line list (7/27, 26%), notifications or alerts (1/27, 4%), registration (1/27, 4%), and reports (1/27, 4%).

## Discussion

### Principal Findings

This pilot evaluation study of the novel FluCARE app found the app to be acceptable, useful, and safe for use by ACF staff, the primary target users of the app. Thus, the app has demonstrated feasibility for ongoing implementation and further scale-up to other public health districts. User acceptance and satisfaction are fundamental to the long-term success of an app, particularly for adoption and sustained use [28]. FluCARE was highly acceptable to ACF staff, with a majority of users indicating that they would definitely use the app for future outbreaks and that they would definitely recommend the app to others. This is consistent with the high user rating of FluCARE, with an above average overall score (3.91 out of 5) and above average to excellent ratings in functionality, aesthetics, and information quality. There was unanimous agreement in the overall utility of FluCARE in assisting ACFs in outbreak management. Specifically, ACF staff agreed that the app improved outbreak recognition and reduced time to notification of the PHU and response stakeholders. Although these were not objectively measured with outbreak data, we have the ability to collect and report on these data (as shown in Table 2) in future efficacy and effectiveness studies. Finally, there were no adverse events reported in the use of FluCARE, particularly with regards to missed outbreaks, indicating that the app algorithms were working in accordance with national guidelines. Rather than any intrinsic app issues, the predominant reported issues were related to site access, having been addressed with improvements in the hosting environment.

Since the pilot period for FluCARE, there have been at least two instances of specific mobile health (mHealth) apps designed to help detect, manage, and report on respiratory outbreaks in institutions here in Australia [29] and overseas [30]. The article from Ahn et al [29] describes the development of their app, noting that an mHealth app with dashboard functionality should help improve collection and reporting of outbreak data. However, although the article from Echeverria et al [30] describes the implementation and use of their mHealth app, including the detailed collection of line listing data from over 196 institutions and 10,000 sick residents (albeit only for 30 days), their article does not mention any evaluation focused on the implementation barriers or enablers from an organizational or workforce perspective.

This study also provided findings on barriers and facilitators for ACF staff in using FluCARE, which informs a further roll-out of FluCARE. It is well-recognized that an effective and sustainable digital health intervention is dependent not only on the app or digital tool itself but also on various implementation support to ensure an enabling environment for the adoption and use of the digital tool [31]. Our study found that the implemented training and education for FluCARE and the technical support from the PHU were effective and useful in facilitating FluCARE use by ACF staff. This is consistent with previous reviews of eHealth implementation, which found that adequate staff training (including allocation of time and resources for training) and dedicated technical support are critical to increased user acceptance [32,33]. These elements also help to overcome a number of common barriers, such as negative attitudes to a new technology, skill deficits, and disruptions to existing workflow [32,33]. Similarly, as shown in our study, evidence has demonstrated that training of ACF staff and provision of 24/7 support are essential for any implementation of new health technology in nursing homes. Support can be delivered in various formats, such as troubleshooting guides and telephone help lines [34]. In our study, identified barriers for ACF staff to use FluCARE were (1) access to FluCARE outside the organization, (2) lack of involvement in the design of the app, and (3) accountability and lack of incentive within their organization to use the app. Addressing these barriers will require a combination of technical, social, and organizational approaches. For example, in addition to the prior use of consultative processes and ACF engagements [21], establishing direct feedback mechanisms (eg, via the app), including ACF staff in the multidisciplinary FluCARE working group, and training staff champions to lead implementation in each facility are potential strategies. Involving end users in design and development and empowering nursing staff to plan and lead implementation in their facility reduce barriers related to user-friendliness; compatibility (to work environment or processes); and acceptance, confidence, and ownership of the new technology [31,33]. Further exploration of whether FluCARE can integrate with clinical applications to streamline notifiable disease management in collaboration with local geriatric specialist colleagues is needed.

Last, to our knowledge, this is the first time a web-based app for outbreak management in ACFs in Australia has been evaluated and reported in a feasibility pilot program. This study contributes a structured, replicable approach to a feasibility assessment of a novel app in providing the necessary evidence base to guide further implementation and scale-up.

### Limitations

A key limitation of this study is the fundamental alteration in the public health context and respiratory disease transmission dynamics due to the COVID-19 pandemic, the onset of which coincided with the latter half of the study period. This was a period of extraordinary public health measures against COVID-19, including closure of Australia’s border, which also saw an all-time low in circulating influenza and other respiratory viruses [35]. Consequently, none of the participating ACFs experienced an influenza outbreak in which FluCARE could be used, although ACFs still actively used FluCARE for influenza and COVID-19 case monitoring. It is unclear the direction of impact the concurrent pandemic may have had on ACF staff perceptions of FluCARE. Heightened concern for timely detection of respiratory infections may have promoted FluCARE utility. Simultaneously, increased PHU oversight and support of ACFs for COVID-19 may have superseded some of FluCARE’s functions, thus reducing its perceived utility over this period. Another limitation of this study was that ACF participation in the FluCARE pilot and staff participation in the evaluation study were self-selected. This may have resulted in selection bias toward facilities and staff who have pre-existing openness or confidence for new technologies, thus bolstering the acceptance of FluCARE.

This study took place in a single district within metropolitan Sydney. Therefore, findings may not be generalizable to all other public health jurisdictions, such as regional or rural areas with lower densities of ACFs and different levels of digital infrastructure. Further implementation research is necessary to inform and adapt strategies for roll-out into other districts.

Our study was limited to using an evaluation instrument designed to measure the acceptability and safety of mobile-based apps at the time, as no other validated tools were available to evaluate web-based apps specifically. In addition, piloting our ACF user survey enabled the instrument to reach face and content validity; further work is required to ensure the survey has adequate internal reliability over time. Additionally, this study only reports on the perceptions of the ACF staff. Although they are the primary target users of the app, there are also PHU users and other stakeholders whose perceptions are relevant for app adoption and utilization. Further evaluation surveys should also include perceptions of PHU users and collect and analyze in-depth qualitative data to uncover any insights around adoption and use not already reported.

### Conclusion

FluCARE demonstrates high acceptance, utility, and safety for ACF staff in the management of influenza and COVID-19 outbreaks. FluCARE was piloted with a package of implementation activities including mandatory user training and technical support for ACFs. FluCARE has demonstrated feasibility for efficacy studies including further scale-up to other districts. Further evaluation, guided by implementation science frameworks and utilization of outbreak metrics, will be needed.

## Abbreviations

ACF: aged care facility
FluCARE: Influenza Outbreak Communication, Advice and Reporting
ICT: Information Communication Technology
ILI: influenza-like illness
mHealth: mobile health
NSW: New South Wales
PHU: public health unit
SLHD: Sydney Local Health District
uMARS: user Mobile Application Rating Scale
